# Genetic mapping of metabolic traits in the blind Mexican cavefish reveals sex-dependent quantitative trait loci associated with cave adaptation

**DOI:** 10.1186/s12862-021-01823-8

**Published:** 2021-05-21

**Authors:** Misty R. Riddle, Ariel Aspiras, Fleur Damen, Suzanne McGaugh, Julius A. Tabin, Clifford J. Tabin

**Affiliations:** 1 Department of Biology, University of Nevada, Reno, Reno, NV 89557 USA; 2 Department of Genetics, Blavatnik Institute, Harvard Medical School, Boston, MA 02115 USA; 3 Department of Stem Cell and Regenerative Biology, Harvard University, Cambridge, MA 02138 USA; 4 Ecology, Evolution, and Behavior, University of Minnesota, Saint Paul, MN 55108 USA

**Keywords:** *Astyanax mexicanus*, Cavefish, Quantitative trait loci, Blood glucose, Sex, Metabolism, Liver, Gut, Body condition, Teleost, Evolution

## Abstract

**Background:**

Despite a longstanding interest in understanding how animals adapt to environments with limited nutrients, we have incomplete knowledge of the genetic basis of metabolic evolution. The Mexican tetra, *Astyanax mexicanus*, is a species of fish that consists of two morphotypes; eyeless cavefish that have adapted to a low-nutrient cave environment, and ancestral river-dwelling surface fish with abundant access to nutrients. Cavefish have evolved altered blood sugar regulation, starvation tolerance, increased fat accumulation, and superior body condition. To investigate the genetic basis of cavefish metabolic evolution we carried out a quantitative trait loci (QTL) analysis in surface/cave F2 hybrids. We genetically mapped seven metabolism-associated traits in hybrids that were challenged with a nutrient restricted diet.

**Results:**

We found that female F2 hybrids are bigger than males and have a longer hindgut, bigger liver, and heavier gonad, even after correcting for fish size. Although there is no difference between male and female blood sugar level, we found that high blood sugar is associated with weight gain in females and lower body weight and fat level in males. We identified a significant QTL associated with 24-h-fasting blood glucose level with the same effect in males and females. Differently, we identified sex-independent and sex-dependent QTL associated with fish length, body condition, liver size, hindgut length, and gonad weight. We found that some of the genes within the metabolism QTL display evidence of non-neutral evolution and are likely to be under selection. Furthermore, we report predicted nonsynonymous changes to the cavefish coding sequence of these genes.

**Conclusions:**

Our study reveals previously unappreciated genomic regions associated with blood glucose regulation, body condition, gonad size, and internal organ morphology. In addition, we find an interaction between sex and metabolism-related traits in *A. mexicanus.* We reveal coding changes in genes that are likely under selection in the low-nutrient cave environment, leading to a better understanding of the genetic basis of metabolic evolution.

**Supplementary Information:**

The online version contains supplementary material available at 10.1186/s12862-021-01823-8.

## Background

**Fig. 1:**
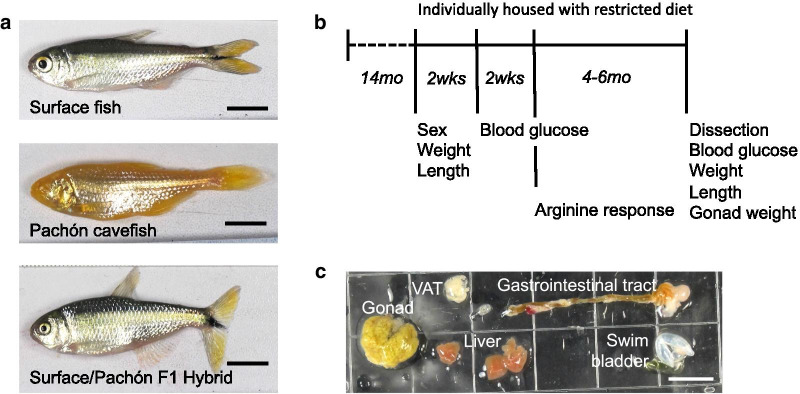
Genetic mapping of metabolism-related traits in *Astyanax mexicanus*. **A** Surface fish and Pachn cavefish were paired to produce F1 surface/Pachn hybrids. F2 hybrids were generated through interbreeding F1 hybrid siblings. **B** Hybrids were raised in group housing to 14months at which point they were housed individually in 1.5L tanks and fed 6mg of pellet food per day for 4months. Figure indicates the timeline for phenotype quantification. **C** Representative image of female F2 organs that were used to measure gastrointestinal tract length, visceral fat area (VAT), and liver area

The Mexican tetra (*Astyanax mexicanus*) is a species that consists of river-dwelling surface fish and eyeless cavefish that evolved in starkly different environments (Fig.1A). Surface fish in rivers from Southern Texas to Central Mexico consume a diet of insects and plants, while cavefish inhabiting perpetually-dark limestone caves in the Sierras of Northeastern Mexico depend predominantly on bat droppings and material brought in by seasonal floods [1]. Some independently evolved cavefish populations (e.g. Tinaja, Molino, Pachn) have converged on similar metabolic adaptations that may be advantageous in caves such as starvation resistance [2, 3], binge-eating (hyperphagia) [3], increased fat accumulation [4], and insulin resistance [5].

The genetic basis of cavefish metabolism has been examined using candidate gene approaches and genomic data to find evidence of cave-specific mutations. For example, hyperphagia and insulin resistance have been associated with genes that are known to regulate appetite and blood glucose in other animals: *melanocortin receptor 4* (*mc4r*) and *insulin receptor alpha* (*insra*) [3, 5]. Comparing gene expression in metabolic tissues such as the liver across wild and lab-raised surface fish and cavefish revealed that environment and phylogeny impact gene activity [6, 7]. The surface fish and cavefish liver transcriptome differs significantly in expression of genes related to carbohydrate metabolism, gluconeogenesis, and glycogen synthesis [6]. Identifying the genetic changes that underlie these shifts in metabolism-related genes is challenging with transcriptome data alone as gene expression differences could be the result of *cis*- or *trans*-regulatory evolution.

A major strength of *A. mexicanus* as a model system is the ability to produce surface/cave F1 hybrids (Fig.1A) that can be crossed to perform quantitative trait loci (QTL) studies to associate genomic regions with trait evolution. Such studies in *A. mexicanus* have focused on morphological and behavioral traits and laid the foundation for functional analyses. For example, a QTL associated with albinism revealed that separate deletions in *oculus albinism 2* (*oca2*) underlie pigment loss in independent cavefish populations [8]. A QTL associated with eye size led to the discovery that cis-regulatory changes that impact expression of *cystathionine beta-synthase* (*cbsa*) contribute to cavefish eye degeneration [9].

Here we present the results of a genetic mapping study focused on metabolic evolution in *A. mexicanus*. We analyzed blood glucose regulation, changes in weight, and internal organ morphology in F2 surface/Pachn hybrids shifted from eating ad libitum to a fixed amount. We found that female F2s are larger than males and have a bigger liver, longer hindgut, and heavier gonad even after normalizing the values to fish size. Regardless of sex, we observed that small F2 fish tend to gain weight on a nutrient restricted diet, and that weight gain is associated with a bigger gonad and longer hindgut. We used a linkage map with high marker density to identify a significant QTL associated with blood glucose level. We also identified sex-independent and sex-dependent QTL associated with body condition, liver size, relative hindgut length, and relative gonad weight. We found genes within the QTL that show exceptional divergence in Pachn cavefish, suggesting they may be important for cave adaptation. In addition, we report predicted nonsynonymous changes in these genes using sequencing data from wild caught surface fish and Pachn cavefish. Overall, our study reveals genomic regions associated with evolution of metabolism-related traits in cavefish. Combined with functional studies, these findings will further improve our understanding of how cavefish thrive in the unique cave environment.

## Results

### Sex bias in weight and organ size but not blood glucose regulation

We measured metabolism-related traits in surface/Pachn F2 hybrids that were individually housed and consumed 6mg of pellet food per day for a period of 4months (Fig.1B). We found that most traits are significantly different between sexes (Table 1). At the start of the feeding trial, on average, females were significantly bigger than males (average length=5.18cm versus 4.38cm, p=2.1010^13^ False discovery rate (FDR) p-value t-test, average start weight=3.53g versus 1.58g, p=1.0810^19^ FDR adjusted Wilcox). Females also had higher body condition factor (average 2.35 versus 1.83, p=4.0310^18^ FDR Wilcox), which is a standard measurement of fish nourishment level [10]. During the feeding trial, males gained weight and females lost weight (average weight change=0.14g male, 0.23g female, p=5.6010^5^ FDR Wilcox). This equated to an average 14.70% weight increase in males and 0.13% increase in females (p=4.9910^6^ FDR Wilcox). However, on average, females remained significantly larger than males and had better body condition at the end of the trial (average weight 3.33g versus 1.73g, p=1.0710^18^ FDR Wilcox, average length 5.10cm vs 4.43cm, p=4.0910^12^ FDR t-test, body condition 2.38 versus 1.92, p=2.2110^15^ FDR Wilcox). Females have a significantly larger gonad compared to males (average gonad weight=0.42g versus 0.06g, p=1.2910^24^ FDR Wilcox). This alone does not account for the weight difference between sexes, since the body weight after subtracting the gonad is also significantly greater in females (average 2.91g versus 1.65g, p=4.7210^13^ FDR Wilcox). In addition, gonad weight normalized to body weight is significantly greater in females (average 0.13 versus 0.03, p=1.1410^20^ FDR Wilcox). This is also true for non-hybrid *A. mexicanus*. For example, in 1.5-year-old female fish average gonad weight relative to body weight (surface=0.13, Pachn=0.14) is significantly greater than in males of the same age (surface=0.04, Pachn=0.02, n=5 fish per sex and population, t-test comparing sex: surface p=0.003, Pachn p=0.001). Although F2 hybrid females are larger than males, we did not find evidence of elevated fat content; muscle triglyceride level and the amount of visceral fat surrounding the organs are not different between sexes (Table 1).

**Table 1:** Average values for surface/Pachn F2 traits

Trait	Male	N	female	N	t-test_pval	wilcox_pval
Weight start (g)	**1.58**	**100**	**3.53**	**119**		**1.08E19**
Weight 4month (g)	**1.73**	**95**	**3.33**	**115**		**1.07E18**
Length start (cm)	**4.38**	**99**	**5.18**	**117**	**2.10E13**	
Length end (cm)	**4.43**	**91**	**5.10**	**108**	**4.09E12**	
Body condition start	**1.83**	**97**	**2.35**	**116**		**4.03E18**
Body condition end	**1.92**	**89**	**2.35**	**106**		**2.21E15**
Weight change (g)	**0.14**	**96**	**0.23**	**117**		**5.60E05**
% weight change	**14.70**	**95**	**0.13**	**115**		**4.99E06**
Gonad weight (g)	**0.06**	**80**	**0.42**	**100**		**1.29E24**
Weight minus gonad (g)	**1.65**	**81**	**2.91**	**100**		**4.72E13**
Gonad weight/weight	**0.03**	**80**	**0.13**	**60**		**1.14E20**
Blood glucose start (mg/dL)	41.10	48	41.23	111		7.68E01
Blood glucose end (mg/dL)	60.76	84	59.28	84		4.13E01
Blood glucose change (mg/dL)	13.41	70	7.55	105		2.68E01
Arginine blood glucose (mg/dL)	58.46	88	61.26	104		7.08E01
Arginine response	19.97	69	23.93	50	6.25E01	
Muscle triglyceride	0.19	83	0.20	102		7.67E01
Visceral fat area (cm^3^)	0.21	88	0.27	108		7.67E01
Liver area (cm^3^)	**0.31**	**88**	**0.50**	**108**		**6.67E12**
Midgut length (cm)	**1.43**	**83**	**1.66**	**102**	**2.73E05**	
Hindgut length (cm)	**0.64**	**87**	**0.78**	**107**		**6.77E08**
Liver area/fish length	**0.07**	**87**	**0.10**	**107**	**7.36E10**	
Midgut length/fish length	0.32	70	0.33	84	5.00E01	
Hindgut length/fish length	**0.14**	**44**	**0.15**	**44**	**3.20E02**	

Traits that are significantly different between sexes in bold. Students t-test used for normally distributed traits and Wilcox test used for non-normally distributed traits based on ShapiroWilks test. Adjusted false discovery rate p-value is shown [65]

We examined the morphology of internal organs that play essential roles in metabolism: the liver, midgut, and hindgut. Since larger fish have bigger organs, we compared values relative to standard fish length. We found no difference in relative midgut length (average 0.32 male versus 0.33 female, p=0.50 FDR Wilcox), but the relative size of the liver and length of the hindgut are significantly greater in female F2 hybrids (Table 1, normalized liver=0.07 male versus 0.10 female, p=7.3610^10^ FDR Wilcox, normalized hindgut=0.14 male versus 0.15 female, p=0.03 FDR Wilcox). These results reveal a previously unappreciated sex bias in organ size in *A. mexicanus*.

We did not observe a significant difference between male and female 24-h fasting blood glucose levels in surface/Pachn F2 hybrids (Table 1). Overall, blood glucose levels increased after four months on the nutrient restricted diet (starting blood glucose: min=23mg/dL, max=82mg/dL, mean=41.17mg/dL, ending blood glucose: min=23mg/dL, max=120mg/dL, mean=59.92mg/dL). On average, male blood glucose levels increased more than females but not significantly (average 13.41mg/dL male, 7.55mg/dL female, p=0.27 FDR Wilcox). To probe the dynamics of blood glucose regulation, we measured blood glucose levels 5h after F2 hybrids were injected with arginine. Arginine injection stimulates release of insulin and results in a drop in blood sugar in surface fish, but does not change blood sugar significantly in cavefish [5]. We did not observe a difference between male and female blood glucose levels in response to arginine injection (Table 1).

### Correlations between metabolism-related traits

**Fig. 2:**
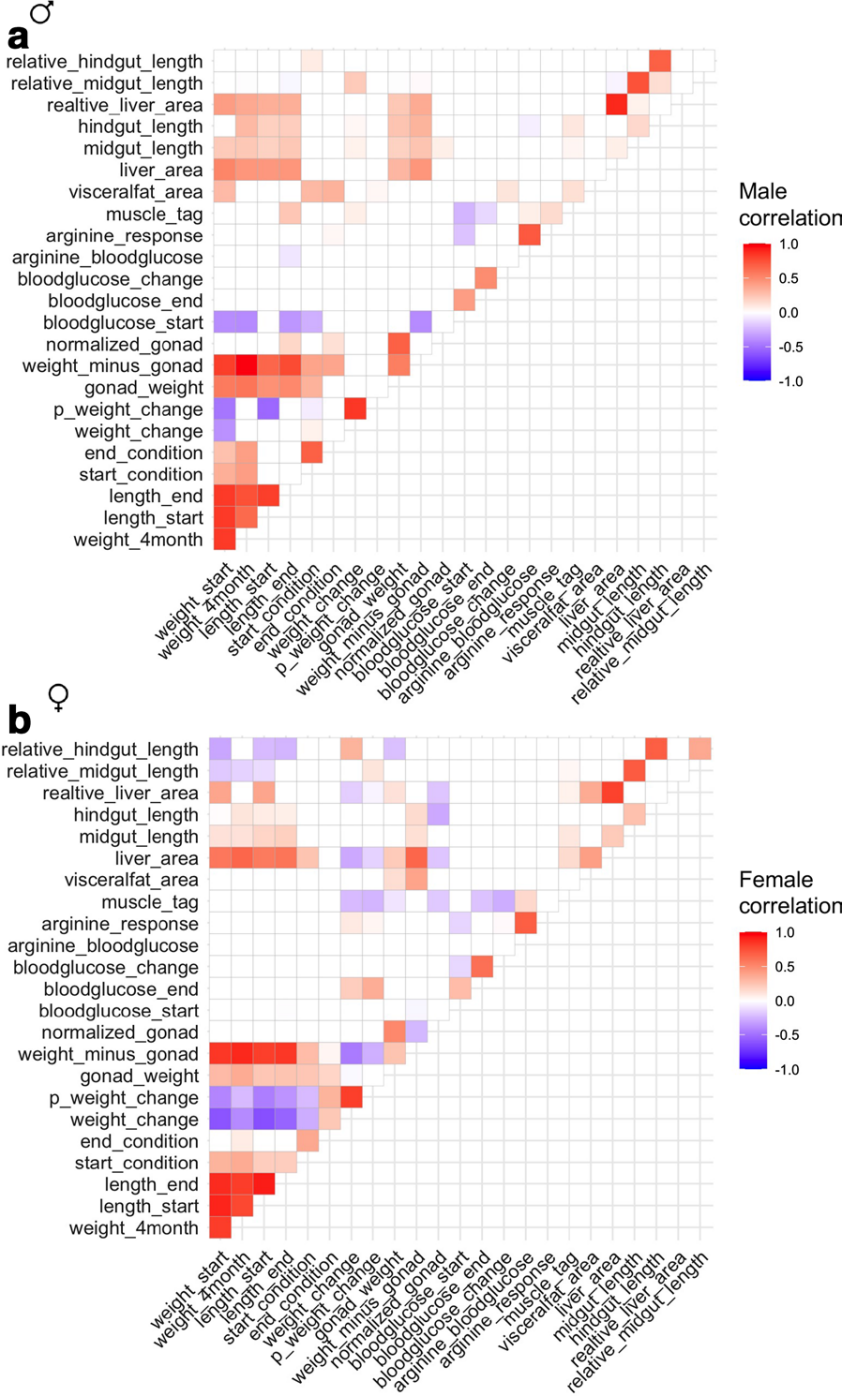
Correlation matrix of metabolism-related traits in *Astyanax mexicanus* surface/Pachn F2 hybrids shifted to a nutrient restricted diet. Kendall rank correlation matrix for traits in F2 hybrid males (**A**) and females (**B**). Color (only shown for significant comparisons) indicates strength and direction of correlation. Percentage weight change (p_weight_change), muscle triglyceride level (muscle_tag)

**Fig. 3:**
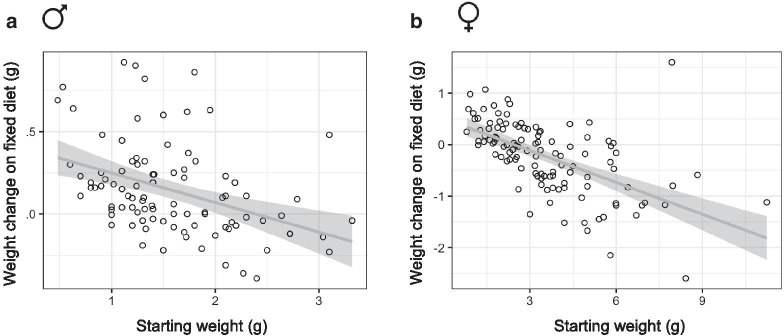
Starting weight correlates with weight change on nutrient restricted diet in *Astyanax mexicanus* F2 hybrids. Scatterplot showing starting weight and weight change after male (**A**) and female (**B**) adult fish were individually housed and consumed a fixed diet for 4 months. Trendline with 95% confidence interval shown in grey

We examined pairwise correlations between the traits measured in our study to explore which factors are associated with tolerance to a nutrient restricted diet (Fig.2A, B). We found that weight change negatively correlates with starting weight in both sexes (Figs.2, 3A, B). Males and females that were greater than 2g at the start of the feeding trial tended to lose weight and smaller fish tended to gain weight. To explore other variables that contribute to weight maintenance we used Akaikes Information Criterion (AIC) to compare linear models for weight change that included the other traits measured in our study. The model with the lowest AIC value (11.50) suggests that starting weight, gonad weight, and hindgut length are significantly associated with weight change (Table 2). Unlike starting weight, gonad weight and hindgut length had a positive effect on weight change (Estimate=0.83 and 0.52 respectively). In other words, smaller fish tend to gain weight, regardless of sex, and weight gain is associated with a bigger gonad and longer hindgut.

**Table 2:** Linear model fitting for weight change

Term	Estimate	Std.error	Statistic	p.value
(Intercept)	0.17	0.17	0.98	3.30E01
Weight start	**0.38**	**0.03**	**12.25**	**2.18E16**
Gonad weight	**0.73**	**0.15**	**4.91**	**1.10E05**
Blood glucose start	0.0017	0.0021	0.81	4.20E01
Arginine blood glucose	0.0011	0.0012	0.95	3.46E01
Visceral fat area	0.33	0.22	1.48	1.45E01
Hindgut length	**0.52**	**0.20**	**2.63**	**1.14E02**

Significant values in bold

We found that starting and ending blood glucose level are positively correlated (Fig.2, combined male and female p=6.385e07, Kendall). In males, starting blood glucose level negatively correlates with starting and ending weight, length, starting body condition, fish weight minus the gonad, and muscle triglyceride level (Fig.2A). In other words, larger, fatter males have lower fasting blood sugar levels. Fasting blood glucose level at the end of the 4-month restricted diet is also negatively correlated with muscle triglyceride level, but not any other trait in males (Fig.2A). In females, we observed a weak negative correlation between starting blood glucose level and fish weight subtracting the gonad (Fig.2B). We found a more dramatic positive correlation between ending blood glucose, weight change, and percent weight change (Fig.2B). These results suggest that higher blood sugar is associated with weight gain in females.

We analyzed linear models for ending blood sugar level that included sex and all of the traits measured in our study as variables. Examining the model with the lowest AIC value (429), we found that starting blood glucose level, muscle triglyceride level, and end weight and length are significantly associated with fasting blood sugar level on the nutrient restricted diet (Table 3). Higher body weight and blood sugar level are associated with higher ending blood sugar level (Estimate=11.31, 0.35), while greater muscle triglyceride level and longer length is associated with lower ending blood sugar (Estimate=26.57, 23.99). These results are surprising considering that cavefish have higher blood sugar and higher fat content [3, 5]. Our results suggest that the interaction of cave and surface fish genotypes in F2 hybrids may impact the association of fat content with fasting blood glucose level.

**Table 3:** Linear model for end blood glucose level

Term	Estimate	Std.error	Statistic	p.value
(Intercept)	**125.87**	**32.55**	**3.87**	**3.60E04**
Weight 4month	**11.31**	**5.47**	**2.07**	**4.47E02**
Length end	**23.99**	**9.23**	**2.60**	**1.27E02**
Gonad weight	1.38	10.55	0.13	8.97E01
Arginine blood glucose	0.09	0.08	1.15	2.58E01
Blood glucose start	**0.35**	**0.14**	**2.46**	**1.81E02**
Muscle triglyceride	**26.57**	**11.32**	**2.35**	**2.35E02**
Visceral fat area	1.72	14.93	0.12	9.09E01
Liver area	4.30	16.76	0.26	7.99E01

Significant values in bold

Arginine injection stimulates release of insulin which reduces blood sugar in surface fish [5]. Since cavefish are insulin resistant, arginine injection does not change blood sugar significantly [5]. In F2 hybrids, blood glucose level after arginine injection is positively correlated with muscle triglyceride level in males (Fig.2A, B). The difference between fasting blood glucose and blood glucose after arginine injection (arginine response) is also positively correlated with muscle triglyceride level in males (Fig.2A). In other words, fatter fish have higher blood sugar levels after arginine injection, suggesting a possible association with impaired insulin response, similar to what is observed in cavefish. In females, arginine response is also positively correlated with weight change and percent weight change (Fig.2B). We analyzed a linear model for arginine blood glucose that includes other traits measured in our study as variables and did not observe any significant associations in the model with the lowest AIC value (493, Table 4).

**Table 4:** Linear model for arginine blood glucose

Term	Estimate	Std.error	Statistic	p.value
(Intercept)	28.07	16.50	1.70	9.56E02
% weight change	0.40	0.24	1.66	1.04E01
Gonad weight	23.30	14.34	1.62	1.11E01
Blood glucose start	0.27	0.25	1.06	2.94E01
Muscle triglyceride	30.77	19.68	1.56	1.25E01
Visceral fat area	18.46	26.53	0.70	4.90E01
Liver area	39.58	23.33	1.70	9.65E02

Liver size and gut length positively correlate with fish size in both sexes (Fig.2A, B). We found that the relative size of the liver and relative length of the hindgut are significantly greater in females (Table 1). Relative hindgut length positively correlates with body condition factor at the start of the food-reduction trial in males and weight change in females, further suggesting the length of the hindgut may contributes to weight gain (Fig.2A, B). We analyzed a linear model for hindgut length including sex, fish length, and other traits measured in our study as variables. Analyzing the model with the lowest AIC value (184), we found that sex, fish length, weight change, and midgut length contribute significantly to hindgut length (Table 5). These quantitative traits have a positive association with hindgut length. In other words, a longer hindgut is linked with weight gain on a nutrient restricted diet. Combined, these results reveal that organ size is sexually dimorphic in *A. mexicanus* and that visceral organ morphology is associated with maintaining weight on limited nutrients.

**Table 5:** Linear model for hindgut length

Term	Estimate	Std.error	Statistic	p.value
(Intercept)	**0.17**	**0.08**	**2.04**	**4.32E02**
Sex	**0.08**	**0.03**	**3.18**	**1.75E03**
Length start	**0.07**	**0.02**	**3.55**	**4.92E04**
Weight change	**0.05**	**0.02**	**2.26**	**2.52E02**
Midgut length	**0.10**	**0.03**	**3.17**	**1.79E03**

Significant values in bold

### Genetic mapping of metabolic traits

We calculated genome wide logarithm of the odds (LOD) scores for each trait using a linkage map and genotype-by-sequencing markers generated as part of a previous study [11] (Additional files 1, 2). For traits that were significantly different between sexes, we compared genome wide scans for QTL, including sex as an additive co-variate (accounting for sex differences, but assuming QTL have the same effect in both sexes) and interactive co-variate (assuming that QTL have a different effect depending on sex). We used a permutation test with 1000 iterations to establish LOD significance thresholds for each trait. Significant sex-interacting QTL were identified by comparing the genome wide LOD thresholds when sex is included as an additive versus interactive covariation (see Methods [12]). Table 6 shows the peak LOD score for each trait and model. We observed significant QTL associated with blood glucose level, standard fish length, body condition, weight change, percent weight change, gonad weight, liver area, and hindgut length. Some of these QTL display significant sex-bias as discussed in more detail below.

**Table 6:** QTL mapping of metabolic traits

Trait	N	Covariate	LOD 5%	LOD 10%	LOD peak	PVE	Peak marker LG position	Scaffold	bp position
Blood glucose start	**108**	**None**	**4.22**	**3.81**	**7.16**	**26.31**	**r18170 9 150.9**	**KB871607**	**839,363**
Blood glucose end	**196**	**None**	**3.92**	**3.61**	**5.85**	**12.84**	**r110832 9 151.8**	**KB872022**	**216,703**
Weight start	219	add_int	7.35	6.59	4.03	8.13	r197874 19 36	KB882103	5,600,729
Weight start	219	Int	7.35	6.59	5.86	11.59	r230947 13 151	KB882132	1,682,036
Weight 4month	210	add_int	7.00	6.55	4.01	8.42	r91812 19 64.3	KB871887	492,183
Weight 4month	210	Int	7.00	6.55	5.46	11.28	r230947 13 151.0	KB882132	1,682,036
Length start	216	add_int	5.93	5.41	5.03	10.17	r231495 13 144	KB882132	577,777
Length start	216	Int	5.93	5.41	5.22	10.53	r91812 19 64.3	KB871887	492,183
Length start	**216**	**sex_int**	**2.77**	**2.37**	**3.63**	**7.45**	**r113255 25 90.7**	**KB872047**	**277,233**
Length end	199	add_int	5.75	5.27	4.35	9.58	r197874 19 36	KB882103	5,600,729
Length end	199	Int	5.75	5.27	4.83	10.58	r230947 13 151.0	KB882132	1,682,036
K start	**213**	**add_int**	**4.00**	**3.56**	**4.21**	**8.70**	**r132099 2 35.1**	**KB872277**	**193,967**
K end	**195**	**Add**	**3.98**	**3.68**	**5.65**	**12.49**	**r134839 2 97.6**	**KB872296**	**1,995,763**
K end	**195**	**sex_int**	**2.17**	**1.84**	**3.89**	**8.78**	**r113674 6 107**	**KB872052**	**163,270**
Weight change	213	Int	6.62	6.12	4.8	9.86	r107738 25 87.5	KB871991	413,427
Weight change	**213**	**sex_int**	**3.31**	**2.88**	**3.4**	**7.09**	**r107738 25 87.5**	**KB871991**	**413,427**
% weight change	**210**	**Add**	**4.03**	**3.73**	**4.04**	**8.48**	**r231495 13 144**	**KB882132**	**577,777**
Gonad weight	180	Int	7.96	6.90	5.84	13.88	r5323 1 41.8	KB871579	6,548,071
Gonad weight	180	Int	7.96	6.90	5.65	13.46	r28416 19 75.4	KB871633	1,033,602
Gonad weight	180	Int	7.96	6.90	4.99	11.99	r47899 22 143.9	KB871694	475,774
Gonad weight	**180**	**sex_int**	**3.79**	**3.23**	**3.03**	**7.46**	**r5323 1 41.8**	**KB871579**	**6,548,071**
Weightgonad	181	Int	6.76	6.14	5.3	12.62	r230947 13 151	KB882132	1,682,036
Normalized gonad	140	Int	6.76	6.29	6.25	18.58	r177364 1 76.5	KB882087	2,900,519
Normalized gonad	140	Int	6.76	6.29	5.37	16.19	r104862 22 178.1	KB871966	141,642
Normalized gonad	140	Int	6.76	6.29	5.4	16.27	r1393 24 29.5	KB871578	4,785,777
Normalized gonad	**140**	**sex_int**	**3.26**	**2.97**	**3.63**	**11.26**	**r177364 1 76.5**	**KB882087**	**2,900,519**
Muscle TAG	192	None	4.10	3.70	na				
Visceral fat area	119	None	4.28	3.89	na				
Liver area	185	add_int	6.07	5.64	4.37	10.31	r154446 7 153	KB873184	12,227
Liver area	185	Int	6.07	5.64	5.38	12.53	r230914 13 150.8	KB882132	1,586,871
Liver area	**185**	**Int**	**6.07**	**5.64**	**6.39**	**14.71**	**r28572 19 74.2**	**KB871633**	**467,313**
Liver area	**185**	**sex_int**	**2.93**	**2.50**	**3.16**	**7.56**	**r248014 7 153**	**KB882149**	**2,507,088**
Midgut length	196	sex_int	5.09	4.75	3.43	7.74	r195661 4 145	KB882102	1,812,243
Hindgut length	**196**	**add_int**	**4.05**	**3.65**	**4.64**	**10.33**	**r172341 10 68.3**	**KB882083**	**490,666**
Hindgut length	**196**	**sex_int**	**2.30**	**1.95**	**3.14**	**7.11**	**r33595 6 62.4**	**KB871649**	**380,272**
R liver area	185	add_int	5.81	5.40	5.62	13.06	r154446 7 153	KB873184	12,227
R liver area	**185**	**sex_int**	**2.79**	**2.43**	**2.95**	**7.08**	**r170604 21 12.8**	KB882082	730,436
R midgut length	194	Add	5.23	4.78	na				
R hindgut length	**194**	**add_int**	**5.11**	**4.79**	**4.68**	**10.51**	**r172341 10 68.3**	**KB882083**	**490,666**
R hindgut length	**194**	**sex_int**	**2.29**	**1.88**	**3.83**	**8.69**	**r59663 4 220**	**KB871744**	**301,165**

Significant traits in bold. Number of individuals (N), covariate model (add=additive, int=interactive, sex_int=sex interacting QTL), logarithm of the odds (LOD) significance thresholds, peak marker LOD score, percent variance explained (PVE),peak marker name and position on indicated linkage group (LG) in centimorgans, and position on Pachn cavefish genome scaffold in base pairs (bp). Abbreviations for traits: body condition factor (K), triglyceride (TAG), length relative to standard fish length (R)

### QTL associated with blood glucose level

**Fig. 4:**
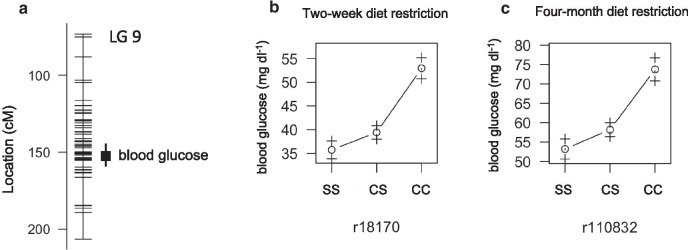
Quantitative trait loci associated with blood glucose level in *Astyanax mexicanus*. **A** Location of markers (horizontal lines) on linkage group 9 indicating the peak position and bayes credible interval (box and whiskers) for QTL associated with 24h fasting blood glucose level. **B** Average fasting blood glucose level (1SE) of F2 surface/Pachn hybrids of the indicated peak linkage group 9 QTL marker genotype after 2-weeks on the nutrient restricted diet (S=surface allele, C=cave allele). **C** Average fasting blood glucose level (+/1 1SE) of F2 surface/Pachn hybrids of the indicated peak linkage group 9 QTL after 4months on the nutrient restricted diet for each genotype (S=surface allele, C=cave allele)

Tinaja and Pachn cavefish carry a coding mutation in *insulin receptor alpha* (*insra* ENSAMX00000001680) that is associated with insulin resistance and weight gain [5]. The mutation is necessary, but not sufficient, for elevated fasting blood sugar levels suggesting that elevated blood sugar in cavefish is likely the result of multiple genetic changes. Consistent with this hypothesis, we found that fasting blood glucose level is associated with significant QTL on linkage group nine of the genetic map used in this study and the *insra* mutation is on linkage group eight (Fig.4A). The peak marker for fasting blood glucose at the start of the feeding trial at 151cM on linkage group 9 has a LOD score of 7.15 accounting for 26% of the phenotype variance. The peak marker for fasting blood glucose at the end of the feeding trial is nearby at 152cM on linkage group 9 with LOD score of 5.85 accounting for 13% of the phenotype variance. As expected, the cave genotypes at the peak markers are associated with the highest blood glucose levels (Fig.4B, C). These findings reveal a genomic location with a strong effect on fasting blood glucose level in *A. mexicanus*.

The peak marker for starting blood glucose maps to scaffold KB871607.1 on the Pachn cavefish genome. To investigate which genes within the QTL may be associated with cave adaptation, we determined several population genetic metrics using the coding regions of all of the genes on this scaffold utilizing whole genome sequencing data from wild caught cavefish and surface fish populations [13]. This dataset includes Tinaja (n=10), Pachn (n=10), and Molino (n=9) cave populations and two surface fish populations: Ro Choy (n=9), which is representative of our lab strains and is more closely related to the stock of fish that invaded the Molino cave, and Rascn (n=8), which is more representative of a separate stock of surface fish that invaded the Tinaja and Pachn caves [13]. We found that one gene on scaffold KB871607.1, *ADAM metallopeptidase domain 9* (*adam9*) exhibits evidence of non-neutral evolution and is therefore likely to be under selection (estimation of hapFLK statistic resulted in 20 p-values less than 0.05 for this gene, Additional file 3). We aligned the predicted cDNA sequence of *adam9* using sequences from the surface fish and Pachn cavefish reference genomes and whole genome sequencing data from wild caught fish [13] and discovered four predicted single amino acid substitutions (F87L, Q384R, D553E, K681N) and one predicted frame shift (T255Ffs, Table 7, Additional file 4). The amino acid substitutions are homozygous and different in all samples while the frame shift is present in 5/10 Pachn cavefish and 2/10 Rio Choy surface fish. *Adam9* has not been associated with blood sugar regulation to our knowledge but investigating the impact of these coding differences represents an avenue for future discoveries. The nearest gene to the peak marker, *nuclear repressor corepressor 2* (*ncor2*), is however a part of a gene network that regulates metabolism [14]. While *ncor2* does not display evidence of non-neutral evolution comparing the coding sequence, there could be regulatory changes that impact *ncor2* expression. Exploring *ncor2* gene regulation and *adam9* gene function represent promising directions for future work on the evolution of cavefish blood glucose regulation.

**Table 7:** Predicted changes to the amino acid sequence based on cDNA alignments of genes within metabolism-related QTL that display evidence of non-neutral evolution by HapFLK analysis and have fixed differences between Ro Choy surface fish and Pachn cavefish (n=10 fish per population)

Trait	Gene name	Predicted surface to Pachn amino acid changes
Blood glucose	*adam9*	F87L, T255Ffs*, Q384R, D553E, K681N
Weight and length	*ano5b*	K67E*, S119L, K639R
Weight and length	*dpep2*	No gene annotated in surface fish genome
Body condition	*ahnak2*	E104K*
Body condition	*exoc3l4*	K431R*, L435M, L483S*, H524Q*, L527E
Gonad weight	*amfr*	Two synonymous differences
Gonad weight	*ano9a*	L42W*, E178D*, E362D, T735I*, A811G*, L744Q*, P749H, V752F*, G757S*, E835K
Gonad weight	*cd44a*	I9M*, Y23_F34del*, Q53_L54insRSCARFWVQHWPV*
Gonad weight	*slc1a2b*	Q215E
Gonad weight	ENSAMXG00000016170	No gene annotated in surface fish genome
Gonad weight	*gzm3*	V28A*, R33L*, E131V, D175S, S196A, T201S, K204T, K216Q*, L226G, T227F
Gonad weight	*si:dkey-100n23.5*	R184del*
Gonad weight	*tnfsf13b*	One synonymous difference
Liver size	*si:ch1073-174d20.1*	A9V, S101N
Liver size	*nfat5a*	D43A, T550N, N611K, Q918K, N1079_Q1083del*, Q1084E*, T1093S, S1293A, I1334M
Hindgut length	*cbf2*	Y389H*, M542T*
Hindgut length	*cbf1*	R27T*, R46Q*, S48P*, V75A*, F101Y*, H142D, E264Q, M266I, K299N, T387I*, E561D, I621N*, K671T*
Hindgut length	*zgc:110782*	E92K
Hindgut length	*gpr119*	No gene annotated for surface fish genome
Hindgut length	*rbm14a*	Two synonymous differences
Hindgut length	*rin1a*	S203N, M464Q*, G768S
Hindgut length	*mark2a*	S392N*, N722K
Hindgut length	*adssl*	F108Lfs*, N224Y, K338R, N367Tfs*, A404Lfs*, C444R
Hindgut length	*spry3*	P3L
Hindgut length	*rce1b*	P116S, I147Lfs*#, F193del*#
Hindgut length	*tenm2*	W411Ffs*
Hindgut length	*zgc:77262*	G288_A291del*, P293_P294del*, G327S
Hindgut length	*lsm14b*	G78C*, A173T*, L266S*
Hindgut length	*fam65b/ripor2*	D468Efs*#, R664H
Hindgut length	*rfx5*	G342R*
Hindgut length	*txnla*	A496T, E504K*, F529C*
Hindgut length	*cpsf1*	L53P*, E355D*, A702T*
Hindgut length	*adck5*	M552I, A569V*

See Additional file 4 for gene and transcript IDs. Asterisk (*) indicates that the change is not homozygous and different between all samples and number sign (#) indicates the change is annotated relative to surface fish reference genome although that is not the most abundant variant in the surface fish samples (see Additional file 4 for details)

### QTL associated with weight, length, and body condition

**Fig. 5:**
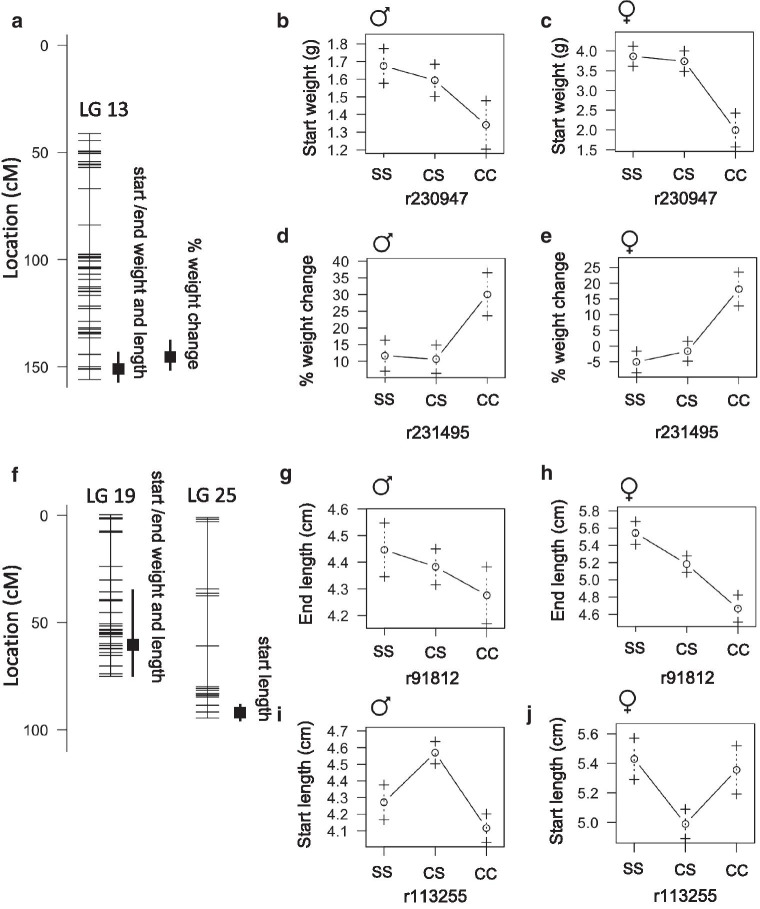
Quantitative trait loci associated with weight, length, and percentage weight change on a nutrient restricted diet in *Astyanax mexicanus*. **A** Location of markers (horizontal lines) on linkage group 13 indicating the peak position and bayes credible interval (box and whiskers) for QTL associated with the indicated trait. **B**, **C** Average weight (1SE) of adult F2 surface/Pachn hybrid females and males of the indicated peak marker genotype (S=surface allele, C=cave allele). **D**, **E** Average weight change as a percentage of total weight (+/1 1SE) of F2 surface/Pachn hybrids of the indicated linkage group 13 peak marker genotype (S=surface allele, C=cave allele, after 4months on a nutrient restricted diet). **F** Location of markers (horizontal lines) on linkage group 19 and 25 indicating the peak position and bayes credible interval (box and whiskers) for QTL associated with the indicated trait. **G**, **H** Average standard length (1SE) of adult F2 surface/Pachn hybrid females and males of linkage group 19 peak marker genotype (S=surface allele, C=cave allele). **I**, **J** Average standard length (1SE) of adult F2 surface/Pachn hybrid females and males of linkage group 25 peak marker genotype (S=surface allele, C=cave allele)

We found that weight and length at the start and end of the feeding trial are associated with QTL on linkage group 13 and 19 with peak markers in close proximity or at the same position (Fig.5A, F, Table 6). On linkage group 13, start/end weight and end length produce a peak at 151cM accounting for approximately 12% of the variance in each trait. The cave genotype is associated with lower weight and shorter length in both males and females (Fig.5B, C). We found that percent weight change is also associated with a QTL on linkage group 13 at 144cM that accounts for 8% phenotype variance. In this case, the cave genotype is associated with weight gain (Fig.5D, E). The peak markers for the linkage group 13 QTL map to Pachn scaffold KB882132.1. None of the genes on this scaffold exhibit evidence of non-neutral evolution (Additional file 3). On linkage group 19, the peak markers for starting weight, starting length, ending weight, and ending length are at 36cM, 64.3cM, 64.3cM and 62.4cM respectively likely representing the same overlapping QTL. The cave genotypes are associated with lower weight and shorter length in both males and females (Fig.5G, H). The peak markers map to Pachn scaffold KB882103.1 and we found that two genes on this scaffold, *ano5b* (ENSAMXG00000005967) and *dpep2* (ENSAMXG00000005810) exhibit evidence of non-neutral evolution (Additional file 3). The *ano5b* gene encodes anoctamin 5b, a calcium activated chloride channel (zfin.org). Mutations in the human *ANO5* gene are associated with reduced bone mineral density [15]. The *dpep2* gene encodes dipeptidase 2, a protein predicted to have metallopeptidase activity (zfin.org). Morpholino knockdown of *dpep2* reduces body size in zebrafish [16]. We aligned the predicted cDNA sequences of *ano5b* from 10 surface fish and 10 Pachn cavefish and discovered three predicted amino acid substitutions (K67E, S119L, K639R, Table 7, Additional file 4). There is no *dpep2* gene annotated on the surface fish genome, so we were not able to investigate coding changes using available sequencing data.

In addition to the QTL on linkage groups 13 and 19, we found a sex-specific QTL associated with starting length on linkage group 25 with the peak marker accounting for 7.45% of phenotype variance; in males, the heterozygous genotype is associated with the greatest length and, in females, the heterozygous genotype is associated with the shortest length (Fig.5F, I, J). The QTL maps to Pachn scaffold KB872047.1 and none of the genes on this scaffold exhibit evidence of non-neutral evolution. Exploring the impact of the coding changes we discovered in *ano5b* represents a promising future direction for understanding the genetic basis cavefish length and weight evolution.

**Fig. 6:**
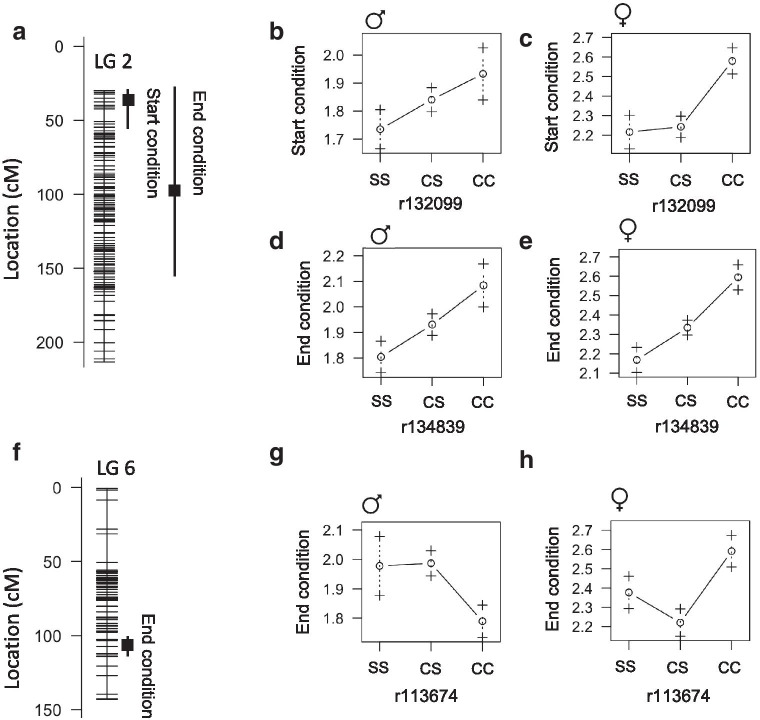
Quantitative trait loci associated with body condition factor in *Astyanax mexicanus*. **A** Location of markers (horizontal lines) on linkage group 2 indicating the peak position and bayes credible interval (box and whiskers) for QTL associated with body condition factor (fish weight (g)נ100/fish length (cm)^3^). **B**, **C** Average body condition factor (1SE) of adult F2 surface/Pachn hybrid females and males of the indicated linkage group 2 peak marker genotype (S=surface allele, C=cave allele, fish fed ad libitum). **D**, **E** Average body condition factor (1SE) of adult F2 surface/Pachn hybrid females and males of the indicated linkage group 2 peak marker genotype (S=surface allele, C=cave allele, fish fed restricted diet for 4months). **F** Location of markers (horizontal lines) on linkage group 6 indicating the peak position and bayes credible interval (box and whiskers) for sex-dependent QTL associated with body condition factor after 4months on a nutrient restricted diet. **G**, **H** Average body condition factor (1SE) of adult F2 surface/Pachn hybrid females and males of the indicated linkage group 6 peak marker genotype (S=surface allele, C=cave allele, fish fed restricted diet for 4months)

Body condition is a measure of fish nourishment level and is a function of length and weight. In the wild, cavefish have better body condition compared to surface fish [17]. We identified QTL associated with body condition that do not overlap the QTL associated with weight and length. Body condition at the start of the feeding trial is associated with a QTL on linkage group 2 with the peak marker at 35.1cM accounting for 8.7% of phenotype variance. End condition is associated with a QTL on linkage group 2 with the peak marker at 97.6cM accounting for 12% of phenotype variance. These are likely the same overlapping QTL and the cave genotypes are associated with better body condition in both males and females (Fig.6AE). The peak marker for end body condition maps to Pachn scaffold KB872296.1. Based on analysis of coding sequences, we found that four genes on this scaffold exhibit evidence of non-neutral evolution and two of the four genes also have fixed differences between Pachn cavefish and both Ro Choy and Rascn surface fish: *ahnak2* (ENSAMXG00000001960) that is predicted to encode a nucleoprotein involved in calcium signaling, and *exoc3l4* (ENSAMXG00000001944) that encodes an exocyst complex component associated with global cortical glucose metabolism in humans [18] (Additional file 3). The single *ahnak2* gene in the Pachn genome is annotated as three separate genes in the surface fish genome. We compared the predicted cDNA sequence from Ro Choy surface fish and Pachn cavefish samples using the surface fish gene that corresponds to the first part of the Pachn gene and discovered a single amino acid substitution (E104K) that is homozygous in all of the Pachn cavefish samples (n=10/10) and half of the Rio Choy surface fish samples (n=5/10, Table 7, Additional file 4). Based on alignments using the same sequencing date we discovered that the Pachn cavefish EXOC3l4 contains five predicted amino acid substitutions (K431R, L435M, L483S, H524Q, L527E, Table 7, Additional file 4). Investigating the impact of the predicted coding changes we observed in *ahnak2* and *exoc3l4* may lead to a better understanding of enhanced body condition in cavefish.

We observed a sex-specific QTL on linkage group 6 associated with end body condition; in males, the cave genotype is associated with lower body condition and, in females, the cave genotype is associated with higher body condition (Fig.6FH). The peak marker maps to Pachn scaffold KB872052.1 and none of the genes on this scaffold exhibit evidence of non-neutral evolution. To our knowledge, *ahnak2* or *exoc3l4* have not been previously implicated in regulating body size, but these genes represent promising candidates for future analysis.

In summary, we found that surface fish genotypes at QTL for weight and length are associated with bigger fish. However, cave genotypes are associated with better body condition and greater weight gain on a limited diet. These genetic differences reflect the growth strategies that evolve in the river versus cave environment.

### QTL associated with gonad weight

**Fig. 7:**
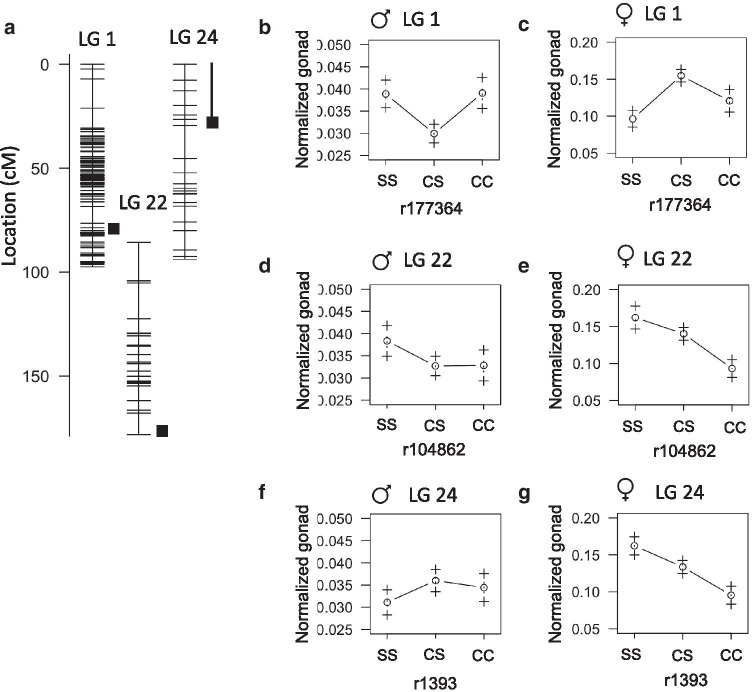
Quantitative trait loci associated with gonad weight in *Astyanax mexicanus*. **A** Location of markers (horizontal lines) on linkage group 1, 22, and 24 indicating the peak position and bayes credible interval (box and whiskers) for QTL associated with the weight of the gonad normalized to fish weight. **BG** Average normalized gonad weight (1SE) of adult F2 surface/Pachn hybrid females and males of the indicated linkage group peak marker genotype (S=surface allele, C=cave allele)

Gonad development in *A. mexicanus* has not been explored, but it is conceivable that the cave environment may have selected for altered reproductive strategies. Cavefish eggs are larger than surface fish eggs and cavefish tend to have smaller clutch sizes [19, 20]. The markers with the highest LOD scores for gonad weight appear on linkage group 1 (41.8cM), 19 (75.4cM), and 22 (143.9cM) and account for 14%, 13%, and 12% of phenotype variance respectively (Table 6). The peak on linkage group 19 is near the QTL for fish length and weight indicating this likely represents the effect of fish size. Normalized gonad weight is also associated with peak markers on linkage group 1 and 22 albeit at different positions (76.5cM and 178.1cM) explaining 19% and 16% of phenotype variance, respectively (Fig.7A). The peak marker on linkage group 1 rises above the LOD threshold for a sex-associated trait and the heterozygous genotype is associated with smaller gonads in males and larger gonads in females (Fig.7B, C). The peak marker maps to Pachn scaffold KB871579.1. We found nine genes on this scaffold exhibit evidence of non-neutral evolution and five of the nine have fixed differences between Pachn cavefish and both river populations: *ano9a* (ENSAMXG00000017855) predicted to encoded a protein involved in calcium activated phospholipid scrambling, *amfr* (ENSAMXG00000017688) that encodes an E3 Ubiquitin Ligase, *cd44a* (ENSAMXG00000017603) that encodes a cell surface receptor, ENSAMXG00000016170 that encodes a NACHT, LRR and PYD domain-containing protein, and *slc1a2b* (ENSAMXG00000017604) which is expressed in the nervous system and is predicted to encode a solute transporter (zfin.org, Additional file 3). CD44 is involved in oocyte maturation in mammals and expressed in ovarian cancer stem cells [21, 22], but a role for CD44 in fish gonads has not been explored to our knowledge. We discovered one predicted amino acid substitution (I9M), an eleven amino acid deletion (Y23_F34del), and a thirteen amino acid insertion (Q53_L54insRSCARFWVQHWPV) comparing the predicted cDNA sequence of *cd44a* between Ro Choy surface fish and Pachn cavefish (Table 7, n=10 fish per population). Using the same sequencing data, we found 10 predicted amino acid substitutions in Pachn Ano9a (L42W, E178D, E362D, T735I, A811G, L744Q, P749H, V752F, G757S, E835K, Table 7, Additional file 4), one amino acid substitution in Pachn Slc1a2b (Q215E), and two cDNA sequence differences in *amfr* that are predicted to cause synonymous changes. There is no gene on the surface fish genome that corresponds to Pachn cavefish ENSAMXG00000016170. Our results reveal possible genetic changes that contribute to size differences in the gonads of surface and cave-adapted *A. mexicanus*.

We found an additional peak marker associated with normalized gonad weight on linkage group 24 that explains 16% of phenotype variance (Fig.7A). The homozygous surface genotype is associated with the lowest normalized gonad weight in males, and greatest gonad weight in females (Fig.7F, G). The peak marker maps to Pachn scaffold KB871578.1 and three genes on this scaffold exhibit evidence of non-neutral evolution: *gzm3* (ENSAMXG00000002176) that encodes a protein with endopeptidase activity, *si:dkey-100n23.5* (ENSAMXG00000001983) that encodes a G-protein coupled receptor, and *tnfsf13b* (ENSAMXG00000003788) that encodes a cytokine which functions in B cell activation (zfin.org, Additional file 3). We discovered ten predicted amino acid substitutions in Pachn Gzm3 (V28A, R33L, E131V, D175S, S196A, T201S, K204T, K216Q, L226G, T227F) and one predicted single amino acid deletion in Si:dkey-100n23.5 (R184del) by comparing cDNA sequences from Ro Choy surface fish and Pachn cavefish (n=10 fish per population, Table 7, Additional file 4)*.* The Pachn cavefish *tnfsf13b* cDNA sequence contained one base pair change that is predicted to result in a synonymous change. Exploring a link between the genes that contain coding changes and gonad development may reveal the genetic basis of increased egg size and reduced clutch size in Pachn cavefish.

### QTL associated with liver size

**Fig. 8:**
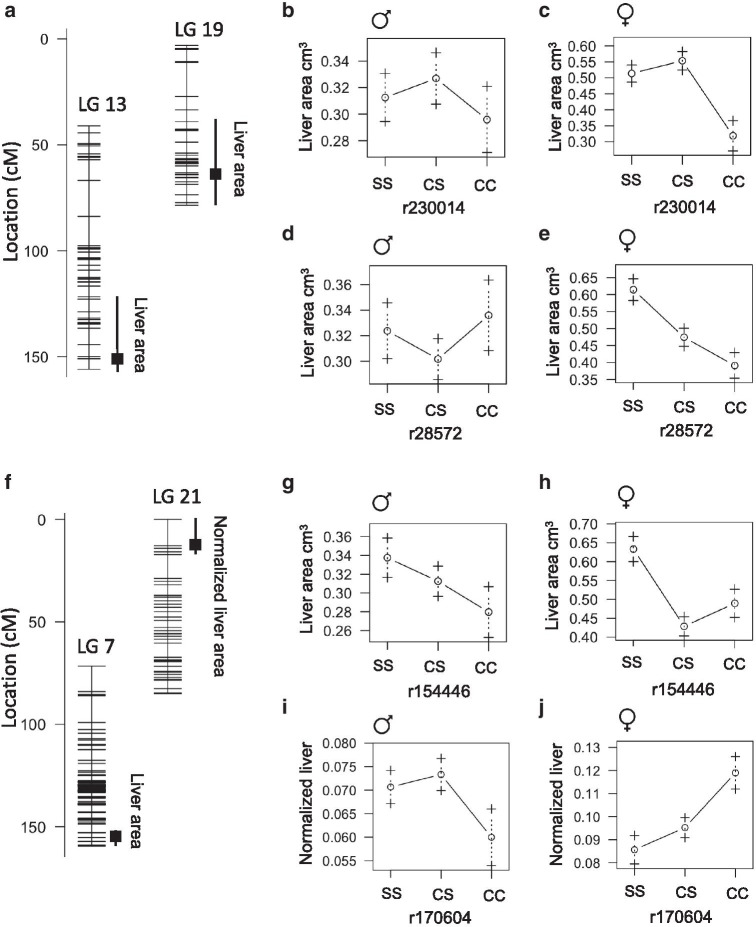
Quantitative trait loci associated with liver area in *Astyanax mexicanus*. **A** Location of markers (horizontal lines) on linkage group 13 and 19 indicating the peak position and bayes credible interval (box and whiskers) for QTL associated with liver area. **B**, **C** Average liver area (1SE) of adult F2 surface/Pachn hybrid females and males of the indicated linkage group 13 peak marker genotype (S=surface allele, C=cave allele). **D**, **E** Average liver area (1SE) of adult F2 surface/Pachn hybrid females and males of the indicated linkage group 19 peak marker genotype (S=surface allele, C=cave allele, fish fed restricted diet for 4months). **F** Location of markers (horizontal lines) on linkage group 7 indicating the peak position and bayes credible interval (box and whiskers) for QTL associated with liver area. **G**, **H** Average liver area (1SE) of adult F2 surface/Pachn hybrid females and males of the indicated linkage group 7 peak marker genotype (S=surface allele, C=cave allele). **I**, **J** Average normalized liver area (1SE) of adult F2 surface/Pachn hybrid females and males of the indicated linkage group 21 peak marker genotype (S=surface allele, C=cave allele)

Cavefish livers are enlarged and accumulate more fat compared to surface fish livers [3]. We found that liver area is associated with a QTL that overlaps with weight and length QTL on linkage groups 13 and 19 (Fig.8A, Table 6, 151cM and 74.2cM accounting for 13% and 15% of phenotype variance, respectively). The heterozygous genotype on linkage group 13 is associated with the largest liver in both males and females (Fig.8B, C). This is contrary to the fish weight/length QTL, for which the homozygous surface genotype is associated with the largest fish (Fig.8B, C). Also, different than the QTL for length on linkage group 19, the cave genotype is associated with greater liver size in males (Fig.8D). We observed an additional QTL for liver size on linkage group 7 at 153cM accounting for 10% of phenotype variance (Fig.8F). The liver size QTL at linkage group 7 is significant and sex specific. In males, the heterozygous genotype is associated with intermediate liver size and, in females, the heterozygous genotype is associated with the smallest liver size (Fig.8G, H). Liver size normalized to fish length is also associated with the same peak marker on linkage group 7 that approaches significance (Table 6). The peak marker maps to Pachn scaffold KB873184.1 and none of the genes on the scaffold exhibit evidence of non-neutral evolution.

We found an additional sex-interacting QTL on linkage group 21 (12.8cM, 7% variance) associated with normalized liver size; the homozygous cave genotype is associated with the largest liver in females and the smallest liver in males (Table 6, Fig.8I, J). The overlap in fish length and liver size QTL on linkage group 13 and 19 suggest that the association between fish size and liver size may contribute to the QTL. However, the QTL on linkage group 7 and 21 may be specifically associated with sexual dimorphism of the liver. The peak marker on linkage group 21 maps to Pachn scaffold KB882082.1; five of the genes on this scaffold exhibit evidence of non-neutral evolution and two have fixed differences between Pachn cavefish and both surface fish populations: *nfat5a* (ENSAMXG00000013845) that encodes a transcription factor involved in immune response [23], and *dbndd1* (ENSAMXG00000013840) a gene that is mutated in humans with HermanskyPudlak Syndrome 7 which is characterized by hypopigmentation and lysosomal storage defects [24] (Additional file 3). We discovered eight predicted amino acid substitutions (D43A, T550N, N611K, Q918K, Q1084E, T1093S, S1293A, I1334M) and a five amino acid deletion (N1079_Q1083del) in Nfat5a, and two amino acid substitutions in Dbndd1 comparing predicted cDNA sequences between Ro Choy surface fish and Pachn cavefish (n=10 fish per population, Table 7, Additional file 4). Investigating the impact of the coding changes may lead to insights into sex- and morphotype-specific liver development.

### QTL associated with length of the hindgut

**Fig. 9:**
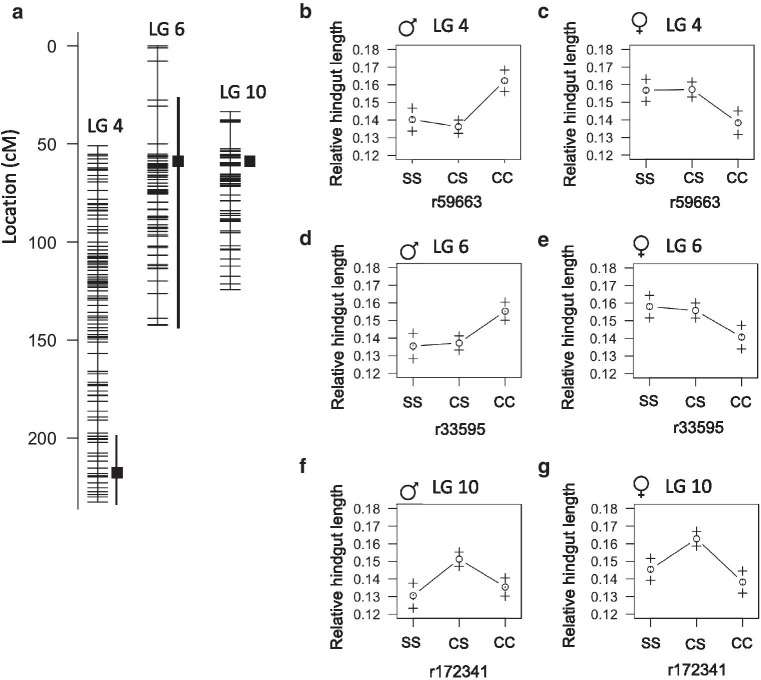
Quantitative trait loci associated with hindgut length in *Astyanax mexicanus*. **A** Location of markers (horizontal lines) on linkage group 4, 6, and 10 indicating the peak position and bayes credible interval (box and whiskers) for QTL associated with relative hindgut length. **BG** Average relative hindgut length (1SE) of adult F2 surface/Pachn hybrid females and males of the indicated linkage group peak marker genotype (S=surface allele, C=cave allele)

We found that hindgut length and relative hindgut length are associated with a QTL on linkage group 10 at 68.3cM accounting for 10% of phenotype variance (Fig.9A, Table 6). The heterozygous genotype is associated with the longest hindgut length in both males and females (Fig.9F, G). The homozygous cave genotype is associated with a longer hindgut in males and shorter hindgut in females compared to the homozygous surface genotype (Fig.9F, G). The peak marker maps to scaffold KB882083.1 on the Pachn cavefish genome and we found 16 genes on this scaffold exhibit evidence of non-neutral evolution (Additional file 2). Twelve of these genes have fixed differences in the coding regions comparing Pachn cavefish to both Ro Choy and Rascn surface fish populations (Tables 7, 8). Two genes are predicted to encode complement factor B-like proteins that are involved in recognizing and eliminating bacteria (herein referred to as *cfb1* and *cfb2*). We identified thirteen predicted amino acid substitutions in Pachn cavefish Cfb1 (R27T, R46Q, S48P, V75A, F101Y, H142D, E264Q, M266I, K299N, T387I, E561D, I621N, K671T) and three predicted amino acid substitutions in Pachn Cfb2 (Y389H, M542T) comparing cDNA sequences from Ro Choy surface fish and Pachn cavefish (n=10 fish per population, Table 7, Additional file 4). Changes to *cfb1/cfb2* may reflect adaptation to the cave microbial landscape [25].

**Table 8:** Hindgut QTL candidate genes

Gene ID	Gene name	Divergence from Rio Choy			Divergence from Rascn		
		Pachn	Tinaja	Molino	Pachn	Tinaja	Molino
ENSAMXG00000007067	*gpr119*	0.71	0.44	0.53	0.41	0.1	0.53
ENSAMXG00000007167	*cbf2*	0.81	0.93	0.65	0.79	0.93	0.65
ENSAMXG00000007196	*cbf1*	0.95	0.94	0.93	0.94	0.94	0.93
ENSAMXG00000007233	*rbm14a*	0.29	0.1	0.11	0.18	0.01	0.11
ENSAMXG00000007243	*rin1a*	0.47	0.3	0.18	0.22	0.12	0.18
ENSAMXG00000007297	*mark2a*	0.2	0.27	0.23	0.32	0.34	0.23
ENSAMXG00000007312	*zgc:110782*	0.82	0.38	0.32	0.79	0.29	0.32
ENSAMXG00000007379	*adssl*	0.86	0.85	0.11	0.47	0.5	0.11
ENSAMXG00000007382	*rce1b*	0.85	0.76	0.72	0.81	0.76	0.72
ENSAMXG00000007446	*tenm2*	0.47	0.47	0.56	0.11	0.13	0.56
ENSAMXG00000025170	*spry3*	0.13	0.3	0.49	0.5	0.39	0.49
ENSAMXG00000025173	*zgc:77262*	0.73	0.55	0.58	0.25	0.05	0.58
ENSAMXG00000002865	*lsm14b*	0.82	0.60	0.61	0.82	0.69	0.47
ENSAMXG00000011121	*fam65b*	0.65	0.73	0.56	0.67	0.72	0.62
ENSAMXG00000011858	*Txnla*	0.64	0.50	0.29	0.73	0.45	0.60
ENSAMXG00000008444	*adck5*	0.42	0.26	0.28	0.29	0.14	0.35
ENSAMXG00000008359	*rfx5*	0.24	0.63	0.29	0.82	0.60	0.73
ENSAMXG00000008485	*cpsf1*	0.22	0.20	0.16	0.23	0.12	0.21

Shown in the table are the proportion of genes in the genome falling below the specified gene for D_xy_ when comparing indicated surface and cave populations. Some gene IDs from Pachn cavefish genome Ensembl database version 89 may be depreciated in most current Ensembl database. See Additional file 4 for updated gene IDs

Genes with high divergence rank in only Pachn cavefish compared to Ro Choy surface fish include *zgc:77262 Lark-like RNA binding protein* (ENSAMXG00000025173), *Zgc:110782* (ENSAMXG00000007312)*,* which has similarity to *prostaglandin F synthase*, and *G-protein coupled receptor 119* (*gpr119,* ENSAMXG00000007067) (Table 7). Each of these genes has reported roles in the gut: the Lark RNA binding protein controls infection-induced RNA splicing [26], prostaglandins protect the gastrointestinal mucosal barrier from noxious chemicals [27], and GPR119, a glucose-dependent insulinotropic receptor, senses fat and regulates appetite and gut motility via the release of glucagon-like peptide 1 (GLP-1), peptide YY (PYY), and glucose-dependent insulinotropic peptide (GIP) from enteroendocrine cells [28,30]. We discovered one predicted amino acid substitution in the Pachn Lark-like RNA binding protein (E92K) comparing the cDNA sequences from Ro Choy surface fish and Pachn cavefish (n=10 fish per population, Table 7). Using the same sequencing data, we found one predicted amino acid substitution (G327S), a four amino acid deletion (G288_A291del), and two amino acid deletion (P293_P294del) in the Pachn Prostaglandin F synthase-like protein (Table 7, Additional file 4). There is no *gpr119* gene annotated on the surface fish genome. Potential links between gut length and the function of Lark, prostaglandins, or GPR119 have not been previously appreciated. It will be informative to test if these genes impact gut length by controlling proliferation in the intestinal epithelium.

We identified significant sex-specific QTL associated with hindgut length on linkage group four and six (Fig.9A, Table 6). The genotypes at the peak marker positions of the sex-specific QTL account for 9% and 6% of hindgut length variance, respectively. The homozygous cave genotypes are associated with the shortest hindgut length in females and longest hindgut length in males (Fig.9C, E). The peak markers for the sex-dependent QTL on linkage group 4 and 6 map to scaffold KB871744.1 and KB871649.1, respectively. We examined all of the genes on each scaffold and found that seven exhibit evidence of selection via HapFLK; two from linkage group four and five from linkage group six (Table 7). One of the genes on linkage group four, *lsm14b* (ENSAMXG00000002865), has fixed differences in the coding region comparing Pachn cavefish to both Ro Choy and Rascn surface fish and has the highest Pachn/Ro Choy divergence rank among the sex-specific QTL genes that exhibit non-neutral evolution (Tables 7, 8). LSM14B is an RNA binding protein that likely regulates translation. In humans, *Lsm14b* is most highly expressed in the testis, brain, and ovary [31]. In zebrafish, it is highly expressed in the ovary [32]. A role for LSM14B in modulating gut length has not been investigated. However, a link between sex and gut length has been uncovered in the fruit fly, *Drosophila melanogaster*, as gut length is influenced by the sexual identity of intestinal stem cells [33]. We discovered three predicted amino acid substitutions in Pachn Lsm14b (G78C, A173T, L266S) comparing predicted cDNA sequences from Ro Choy surface fish and Pachn cavefish (n=10 fish per population, Table 7, Additional file 4). LSM14B therefore represents a promising candidate for future studies aimed at understanding a link between sex and gut length in *A. mexicanus.*

Five of the genes on scaffold KB871649.1 exhibit evidence of non-neutral evolution via HapFLK and have fixed differences comparing the coding regions between Pachn cavefish and Ro Choy and Rascn surface fish (Tables 7, 8). The gene *fam65b* (also known as *ripor2*) shows the second highest ranked divergence between Pachn cavefish and Ro Choy surface fish. It encodes an inhibitor of the small GTPase RhoA. Protein levels of FAM65B are highest in the duodenum, compared to other human tissues, but a role in the gut has not been investigated, to our knowledge [34]. We discovered one predicted amino acid substitution in Pachn Fam65b (R664H) comparing cDNA sequences between Ro Choy surface fish and Pachn cavefish (n=10 fish per population, Table 7, Additional file 4). Using the same sequencing data, we found that half of the surface fish samples (5/10) contained a seven base pair deletion that is predicted to impact the amino acid sequence, resulting in a frame shift (D468Efs).

Another candidate gene on linkage group 6, *rfx5*, shows high divergence rank in Pachn compared to Ro Choy (Table 8). RFX5 binds to the X box of MHC-II promoters to regulate translation [35, 36]. It is expressed in the developing duodenum in humans and, in adults, is highly expressed in immunomodulatory tissues. Mutations in RFX5 in humans lead to severe immunodeficiency, but, interestingly, mutations in the similar protein RFX6 cause malformations of the gut [37]. We discovered a predicted single amino acid substitution in Pachn Rfx5 (G342R) comparing cDNA sequences from Ro Choy surface fish and Pachn cavefish (n=10 fish per population, Table 7, Additional file 4). Investigating the functional impact of this coding change in Rfx5 and the coding changes we discovered in the other candidates associated with hindgut length will further our understanding of the genetic basis of gut length evolution in *A. mexicanus*. Since hindgut length is sex biased and linked to weight change on a nutrient limited diet, these studies will lead to insights into the connections between sex differentiation and metabolism.

## Discussion

Sex dramatically impacts phenotypes, yet, sex has not been considered as a covariate in *Astyanax* genetic mapping studies (see [8, 38,46]). Female *A. mexicanus* have been reported to be larger than males [47], but no other feature has been noted to be impacted by sex (eye size, melanophore number, relative condition, weight loss, tooth number, peduncle depth, fin placement, anal fin rays, SO3 bone width, thoracic rib number, chemical sense, oxygen consumption [2], feeding posture [48], stress behavior [49], and basal sleep [50]). In this study, we observed a sex bias in standard length, weight, body condition, relative liver size, relative gut length, and relative gonad weight in surface/Pachn F2 hybrid *A. mexicanus.* We found that although females are larger than males, males tend to gain weight on a restricted diet and females lose weight. The physiological basis of this phenomenon requires further investigation, but we hypothesize that a combination of behavioral and hormonal differences between the sexes could be at play. In a group setting, smaller fish (which are mostly male) may experience aggression from larger fish (which are mostly female), obtain less food, and remain smaller. When moved to individual housing, smaller fish may obtain more food than they had been able to obtain in a group setting, and larger fish receive less food. Consistent with this hypothesis we found that regardless of sex, smaller fish gained more weight on the nutrient restricted diet compared to larger fish. However, based on a resident-intruder assay, there are no differences in aggression between male and female *A. mexicanus* [51].

Another possibility for the difference in growth between males and females on the restricted diet is that growth was influenced by environmental space; teleost fishes, including *A. mexicanus* [52], exhibit restricted growth when housed in small spaces possibly due to increased stress hormone levels [53]. In our study, hybrid fish were shifted from being housed as a group in a 37L tank to being individually housed in 1.5L tanks. It is possible that the larger fish (that were mostly female) experienced more stress in this space compared to smaller fish (that were mostly male) which restricted their growth. How the sex-bias in size arises initially when fish are in the same space is unclear but in the majority of fish species that have been examined, females grow larger than males [54]. Our results warrant a more thorough examination of the impact of sex on phenotypes and how sex influences behavior under different contexts in *A. mexicanus*.

We found that the relative size of the liver and relative length of the hindgut are greater in female *A. mexicanus*. In other species, characteristics of the liver and colon are sexually dimorphic. For example, in humans, the weight of the liver is greater in males and liver metabolism differs between sexes, resulting in sex-specific drug metabolism and disease susceptibility [55]. Human females have a longer colon than males and increased risk of developing right-sided colon cancer [56,58]. Sex hormones are linked to variation in these traits in humans although the mechanisms of action are not entirely clear. The details of sex determination and differentiation in *A. mexicanus* have yet to be elucidated. Our observations nevertheless provide an opportunity to investigate the evolution of sexually dimorphic traits that relate to growth and metabolism using this species.

Surface fish are longer and weigh more than cavefish, but cavefish have better body condition and lose weight more slowly when fasted [2, 3, 17]. We identified overlapping QTL associated with length, weight, and percent weight change on a nutrient limited diet. We also identified a QTL associated with body condition. Surface alleles are associated with bigger fish and cave alleles are associated with weight increase on a nutrient limited diet and better body condition. These findings highlight the differences in growth strategies between morphotypes. Cavefish have adapted their metabolism to survive starvation [2, 3], while surface fish have adapted to grow larger, perhaps providing an advantage in hunting, competition, and avoiding predators. Previous QTL mapping studies have identified genomic regions associated with body condition and starvation resistance [47]. However, a limited number of genetic markers and unavailable genome annotations made identifying genes in the region difficult. In addition, sex was not considered a covariate in any previous QTL study in *A. mexicanus* to our knowledge.

The dense linkage map we present, combined with availability of the annotated Pachn cavefish and surface fish genomes and genomic data from wild populations allowed us to identify a short list of candidate genes associated with metabolism-related QTL. We mapped traits to the genome and used population genomic analysis to determine if genes within each region display evidence of selection. Although genetic and phenotypic variation must be present for the trait to be mapped, importantly, it does not necessarily mean that the trait is adaptive. It is possible that traits are neutral and therefore genetic changes underlying the trait would not be identified by searching for genes that show evidence of selection. In line with this, genes that display evidence of selection may not control the mapped trait although they happen to be within the QTL. Establishing a connection between the genes within the QTL, the traits, and cavefish evolution will require functional analysis. We identified a number of coding differences between surface fish and Pachn cavefish in genes within metabolic QTL that can be tested for their impact on protein function and role in cavefish trait evolution.

## Conclusions

*Astyanax mexicanus* cavefish have morphological and metabolic adaptations that allow them to thrive in a harsh environment. Utilizing QTL mapping, we identified genomic regions associated with cavefish metabolic traits. We discovered a QTL with strong effect on blood glucose level, in addition to the previously identified *insra* mutation, highlighting that multiple approaches can lead to insights on the genetic basis of cavefish evolution. We found that a number of traits, including fish size, gonad size, and organ size, are sex-biased in *A. mexicanus*. We identified sex-independent and -dependent QTL associated with these traits and genes within the QTL that exhibit evidence of non-neutral evolution suggesting they may be important for cavefish adaptation. Furthermore, we reveal predicted amino acid changes in the proteins the genes encode. Functionally testing the role of the identified changes will provide a better understanding of the unique survival and reproductive strategies that evolve in a nutrient limited environment.

## Methods

### Biological specimens

*Astyanax mexicanus* surface fish and Pachn cavefish used to generate F2 hybrids were derived from fish collected from the Ro Choy river and Pachn cave in Mexico that have been bred in the laboratory of Clifford Tabin at Harvard Medical School for multiple generations. For population genomic analysis, surface fish were collected from Ro Gallinas (Rascn) and Ro Choy, and cavefish were collected from Pachn, Tinaja, and Molino Caves in Mexico. *Astyanax aenus* were collected in Guerrero, Mexico. Additional details are available as part of a previous study [13].

### F2 hybrid population and trait quantification

F1 surface/Pachn hybrids were generated from paired breeding of a male surface fish with a female Pachn cavefish (Fig.1A). The F2 mapping population (n=219) consisted of three clutches produced from breeding paired F1 surface/Pachn hybrid siblings. Fish were housed at densities of less than one adult per 2L until they were 14months old and then were moved to individual 1.5L tanks and fed three pellets (approximately 6mg) of New Life Spectrum TheraA+ small fish formula once per day for at least 4months and as long as 6months (Fig.1B). Fasting blood glucose was measured using FreeStyle Lite blood glucose meter and test strips 24h after the fish were fed. To measure arginine response, arginine (6.6M/gram fish) was injected into the intraperitoneal cavity using a U-100 insulin needle and blood glucose was measured after 5h. For non-lethal blood glucose measurement, blood was collected from the caudal tail vein using a U-100 insulin needle. For final blood glucose measurement blood was collected from the caudal tail vein after the tail was removed. Fish were euthanized in 1400ppm Tricane. Images were taken using a Cannon Powershot D12 digital camera (Fig.1A, C). Measurements were made using ImageJ. Body condition factor was calculated as fish weight (g)נ100/fish length (cm)^3^ [10].

### Muscle triglyceride quantification

Fillets of skeletal muscle were flash frozen in liquid nitrogen and stored in 80C. Frozen fillets were ground to powder in liquid nitrogen with a mortar and pestle. The muscle powder (100mg/mL) was resuspended with 5% NP-40/ddH20 and slowly heated to 100C for 5min and cooled back to room temperature. The heating and cooling were repeated two times after which the samples were centrifuged at max speed using a microcentrifuge for 2min. The supernatant was then extracted and diluted tenfold with ddH20 before calorimetrically measured using a Triglyceride Assay Kit (Abcam, ab65336).

### Statistical analysis

Statistical analysis and data visualization were performed using R version 3.6.1 [59]. To find the appropriate model for comparing male and female F2 hybrids, correlation analysis, and QTL mapping we first determined if each trait is normally distributed using a ShapiroWilks test. For phenotypes that were normally distributed in both sexes (p>0.05), we tested the impact of sex on each phenotype using a two-sample t-test. For non-normally distributed phenotypes (p<0.05), we used a Wilcoxon signed-rank test. We examined correlations between phenotypes with data subset by sex using Kendall rank test. Correlations were visualized with the ggcorrplot R package version 0.1.3.

### Quantitative trait loci analysis

R/qtl version 1.462 [60] was used to calculate genome wide logarithm of the odds (LOD) scores for each trait using a linkage map and genotype-by-sequencing markers generated as part of a previous study [11]. A single-QTL model and HaleyKnott regression was used to assess statistical significance of LOD scores by calculating the 95th percentile of genome-wide maximum penalized LOD score using 1000 random permutations (scanone). For phenotypes that were significantly different between males and females we compared the results of genome wide scans using sex as an additive and interactive co-variate. Significant sex-by-QTL interactions were identified by comparing the genome wide LOD thresholds according to the methods of [12]. Confidence intervals for identified QTL were estimated using the 95% Bayesian credible interval (bayesint) and 1.5 LOD support interval (lodint) expanded to the nearest genotyped markers. Marker positions on the cavefish genome were determined using the Pachn cavefish assembly (AstMex102, INSDC Assembly GCA_000372685.1, Apr 2013).

### Population genomic metrics and analysis

We performed the following measures with GATK-processed data, including a core set of samples analyzed in previous studies [5, 13] which contained wild caught: Pachn, N=10 (9 re-sequenced plus the reference reads mapped back to the reference genome); Tinaja N=10; Molino N=9; Rascn N=8; and Ro Choy N=9 and required six or more individuals have data for a particular site. Details of sequencing and sample processing are in [5, 13]. We used VCFtools v0.1.13 [61] to calculate , F_ST_ and *d*_XY_ and custom python scripts to calculate these metrics on a per gene basis. We identified the allele counts per population with VCFtools and used these for subsequent *d*_XY_ and fixed differences (Df) calculations. We used hapFLK v1.3 https://forge-dga.jouy.inra.fr/projects/hapflk [62] for genome-wide estimation of the hapFLK statistic of across all 44 *Astyanax mexicanus* samples and two *Astyanax aeneus* samples. For all of these metrics, we only used sites that contained six or more individuals per population and calculated the metric or included the p-values (in the case of hapFLK) for only the coding region of the gene. Genes were included in Table 8 if (a) the comparison between Ro Choy and Pachn resulted in a maximum *d*_XY_=1, suggesting that there was at least one fixed difference between Ro Choy and Pachn and (b) HapFLK resulted in at least one p value less than 0.05, suggesting that the haplotype surrounding the gene exhibited evidence for non-neutral evolution.

### Identification of coding changes

Predicted cDNA sequence alignments were obtained from a variant call format (VCF) file of wild-caught surface fish (n=9, [13]) and Pachn cavefish genomes (reference genome [63] and 9 wild-caught samples [13]) aligned to the surface fish reference genome [64]. VCF tools was used to produce a tab format which was formatted into FASTA with custom python scripts with two sequences per individual to accommodate diploids. Alignments were viewed using CLC sequence Viewer 7.7.1 to identify predicted nonsynonymous changes that are homozygous and different in at least four out of ten surface fish and Pachn cavefish samples (Table 7, Additional file 4). We annotated changes relative to the most common sequence in the surface fish samples unless otherwise indicated in Table 7.

## Supplementary Information

**Additional file 1.** Name and position on linkage group and Pachn reference genome of the QTL markers used in this study.

**Additional file 2.** Phenotype and genotype data for the F2 mapping population.

**Additional file 3:** Population genetic analysis of genes on the scaffold with the peak marker of QTLs identified in this study.

**Additional file 4:** Predicted changes to the amino acid sequence based on cDNA alignments of genes within metabolism-related QTL that display evidence of non-neutral evolution by HapFLK analysis, have differences between Ro Choy surface fish and Pachn cavefish, but are not homozygous and different in all samples (n=10 fish per population). Table indicates number of individuals from each population that are homozygous and heterozygous for the indicated change.

## Data Availability

The linked genotype and phenotype data analyzed in this study is included within the article (Additional files 1, 2). Sequencing data analyzed in this study were submitted to the SRA. Project Accession Number: SRP046999, Bioproject: PRJNA260715.

## Abbreviations

QTL: Quantitative trait loci
LOD: Logarithm of the odds
AIC: Akaikes Information Criterion
